# The role of psychological safety and learning behavior in the development of effective quality improvement teams in Ghana: an observational study

**DOI:** 10.1186/s12913-019-4234-7

**Published:** 2019-06-14

**Authors:** Jordan A. Albritton, Bruce Fried, Kavita Singh, Bryan J. Weiner, Bryce Reeve, Jeffrey R. Edwards

**Affiliations:** 1 Telehealth Services, Intermountain Healthcare, Salt Lake City, Utah USA; 2 Department of Health Policy & Management, The Gillings School of Global Public Health, University of North Carolina at Chapel Hill, Chapel Hill, North Carolina USA; 3 Department of Maternal and Child Health, The Gillings School of Global Public Health, University of North Carolina at Chapel Hill, Chapel Hill, North Carolina USA; 4 Department of Health Services, Department of Global Health, School of Public Health, University of Washington, Seattle, Washington USA; 5 Department of Population Health Sciences, Duke Cancer Institute, School of Medicine, Duke University, Durham, NC USA; 6 Kenan-Flagler Business School, University of North Carolina at Chapel Hill, Chapel Hill, North Carolina USA

**Keywords:** Quality improvement, Teams, Psychological safety, Implementation, Child health, Ghana

## Abstract

**Background:**

As lower-income countries look to develop a mature healthcare workforce and to improve quality and reduce costs, they are increasingly turning to quality improvement (QI), a widely-used strategy in higher-income countries. Although QI is an effective strategy for promoting evidence-based practices, QI interventions often fail to deliver desired results. This failure may reflect a problem with implementation. As the key implementing unit of QI, teams are critical for the success or failure of QI efforts. Thus, we used the model of work-team learning to identify factors related to the effectiveness of newly-formed hospital-based QI teams in Ghana.

**Methods:**

This was a cross-sectional, observational study. We used structural equation modeling to estimate relationships between coaching-oriented team leadership, perceived support for teamwork, team psychological safety, team learning behavior, and QI implementation. We used an observer-rated measure of QI implementation, our outcome of interest. Team-level factors were measured using aggregated survey data from 490 QI team members, resulting in a sample size of 122 teams. We assessed model fit and tested significance of standardized parameters, including direct and indirect effects.

**Results:**

Learning behavior mediated a positive relationship between psychological safety and QI implementation (β = 0.171, *p* = 0.001). Psychological safety mediated a positive relationship between team leadership and learning behavior (β = 0.384, *p* = 0.068). Perceived support for teamwork did not have a significant effect on psychological safety or learning behavior.

**Conclusions:**

Psychological safety and learning behavior are key for the success of newly formed QI teams working in lower-income countries. Organizational leaders and implementation facilitators should consider these leverage points as they work to establish an environment where QI and other team-based activities are supported and encouraged.

## Background

Quality Improvement (QI) is a systematic, data-driven approach to improve the delivery of healthcare services [1]. QI typically consists of teams of individuals working together to identify and test improvements in care processes. Although QI is a widely-used approach in high-income countries, lower-income countries are increasingly turning to QI to improve care quality and reduce cost [2, 3]. Research shows that QI is an effective strategy to increase the utilization of evidence-based practices, but QI interventions often fail to deliver the desired improvements [4–6]. One possible explanation for this failure is that even if QI is an effective strategy, it may not always be well implemented [7].

As the key implementing unit of QI initiatives, teams are critical to the success or failure of QI efforts and other group-level clinical interventions [8, 9]. Studies of large-scale interventions involving many teams show that some QI teams are more effective than others [10]. It is important to note that there are multiple types of teams involved in clinical improvement, including temporary work groups, permanent QI teams, and existing teams focused on patient care. This study focuses on QI teams that were developed and intended to continue working beyond a target intervention period. Unfortunately, the role of the team in QI has largely been overlooked, especially in lower-income countries. Furthermore, much of the existing evidence on QI does not consider measures of QI implementation or relevant antecedents [6, 11].

We address these research gaps in an evaluation of the effectiveness of local, hospital-based QI teams in Ghana. The teams were established as part of *Project Fives Alive!* (PFA), a national, multi-year effort to reduce mortality in children under 5 years of age by improving “the processes of care during pregnancy through the most vulnerable period of labor, delivery, and postnatal care.” [12] We test the ability of the model of work-team learning to explain variation in the degree to which teams implement QI methods [13]. The results will identify leverage points that can be targeted to support QI implementation efforts and to develop a mature healthcare workforce in lower-income countries.

### Conceptual framework

Team effectiveness refers to the degree to which a team meets expectations set by the organization [14]. In QI, team effectiveness is synonymous with QI implementation effectiveness, or the consistency and fidelity with which teams implement QI methods to deliver local improvements in care quality [5]. Highly effective teams rarely manifest by accident [15]. Rather, success is determined by numerous internal and external factors [16, 17].

Although researchers have yet to converge on a single unifying model of team effectiveness, predictors of team effectiveness are often grouped into distinct domains. One domain, antecedent conditions, refers to team-level contextual factors, such as team composition, the skills and abilities of individuals within the team, the resources available to the team, and effective leadership [13, 18–20]. Antecedent conditions are distal predictors that typically influence team effectiveness through mediators. Accordingly, a second domain, team beliefs and attitudes, consists of factors directly affected by antecedent conditions, such as psychological safety, team efficacy, commitment, and values [13, 20, 21]. These factors are fluid characteristics that are common targets for interventions to improve team performance. These mediators act on a third domain, team processes and behaviors [18–20, 22]. Team processes and behaviors, including factors such as conflict management, communication, decision-making, and learning behavior, are typically presented as the most proximal predictors of team effectiveness.

This study applies Edmondson’s model of work-team learning, which includes the following factors from the aforementioned domains: coaching-oriented team leadership and perceived support for teamwork (antecedent conditions); team psychological safety and team efficacy (team beliefs); and team learning behavior (team behaviors) [13]. This study includes each of these factors except team efficacy.

According to Edmondson’s model, the most direct determinant of team effectiveness is team learning behavior [13]. We define team learning behavior as a process of detecting and correcting error [23]. Examples of team learning behaviors include seeking feedback, reflecting on work, and discussing mistakes. In contrast to individual learning, team learning refers to team members’ capacity to engage in genuine “thinking together,” where groups collectively discover insights not attainable individually [24]. Team learning behavior is particularly important when information gathering is central to the goal of the team [13]. Furthermore, learning behavior helps organizations adapt and establish new routines [25, 26].

Psychological Safety refers to the shared belief that the team is a safe place for interpersonal risk taking [13]. High psychological safety reflects a team climate of interpersonal trust and mutual respect. Psychological safety alleviates fear of rejection and supports active participation, creating an environment where team members are more likely to recognize errors and address failures and shortcomings [13]. Although psychological safety has never been explored in this context, a sizeable body of work has demonstrated relationships between psychological safety, learning behavior, and team performance [27]. Psychological safety has been linked to improvements in care processes, creative thinking, and exploratory learning [28, 29]. We expect teams with high psychological safety to engage more enthusiastically and consistently in QI. Thus, we propose hypothesis-1:

- **Hypothesis-1**: Team learning behavior mediates a positive relationship between team psychological safety and QI implementation.

Although team leadership is a complex, multidimensional construct, this study focuses on the degree to which the team has a coaching-oriented leader who guides the work of the team [13, 30]. Leaders shape the beliefs and mindsets of teams [22]. Authoritative, punitive leadership discourages team members from engaging in interpersonal risks, like discussing errors [31]. On the other hand, effective leadership cultivates trust and psychological safety, which enhances team performance [32]. Thus, we propose hypothesis-2:

- **Hypothesis-2**: Team psychological safety mediates a positive relationship between coaching-oriented team leadership and team learning behavior.

We define perceived support for teamwork as the collective degree to which team members feel the organization supports the team’s work and provides adequate resources and information [13, 33]. In addition to directly enabling teamwork, high perceived support may lead team members to believe that the work of the team is important. As a result, team members may develop a greater sense of security and a higher willingness to engage in related tasks. When perceived support is low, team members may feel that the work is unvalued and not worth taking risks. Thus, we propose hypothesis-3:

- **Hypothesis-3**: Team psychological safety mediates a positive relationship between perceived support for teamwork and team learning behavior.

## Methods

### Study setting and sample

PFA was a nationwide program in Ghana to reduce under-5 mortality by developing local QI teams to implement evidence-based practices [12]. The project was funded by the Bill and Melinda Gates Foundation and supported by the Institute for Healthcare Improvement and the National Catholic Health Service of Ghana. This study focuses on PFA-affiliated, hospital-based QI teams from the seven southernmost regions of Ghana. These multidisciplinary teams were formed at the beginning of the PFA program, more than a year before their inclusion in this study. Team leaders held a variety of positions and less than a third were physicians. As part of PFA, teams participated in four regional learning collaborative sessions where they shared experiences with QI, discussed successes and failures, and received additional training in QI methods. Project officers provided assistance and helped teams apply concepts from the learning collaboratives. The ultimate goal of PFA was the development of teams that would successfully drive improvement and also be sustainable after the program support concluded.

The unit of analysis in this cross-sectional, observational study is the team. The final sample includes 122 hospital-based QI teams. Teams included in this study were evaluated by project officers and had team members complete the QI team questionnaire.

Power calculations for SEM are substantially more complicated than power calculations for simple regressions, as the required sample size is a function of model structure as well as the ratio of the number of observed to latent variables. Common rules of thumb exist for determining sample sizes in SEM, but Bollen notes that there are no hard-and-fast rules [34, 35]. However, one calculation suggested a minimum of 113 teams solely for the model structure, a sample size that would allow detection of effect sizes of approximately 0.273 [36]. Previous studies suggest an effect size of 0.25 is not unlikely when dealing with similar team-level latent variables [13, 37].

### Measures and data sources

#### Team-level factors

Team-level factors were measured using QI team questionnaires consisting of 85 questions on multiple topics. Each question used a 7-point Likert response scale from “completely disagree” to “completely agree”. The questionnaires were completed by individual team members at the fourth round of learning collaborative sessions. Project officers asked all learning session participants to complete surveys, resulting in a response rate close to 100%. Team members included physicians, pharmacists, nurses, midwives, administrators, and others. The paper surveys were completed during 13 separate meetings from May 2015 to September 2015, approximately 16 to 18 months after the teams were formed. The survey forms were double-coded by two staff members in Ghana. The confidential responses were matched to teams using a coded participate roster.

We used a subset of items from the QI team questionnaire to measure team leadership (three items), perceived support (four items), psychological safety (five items), and learning behavior (three items) (see Table 1). Team scores for each item were calculated as the average response from respective team members. The McDonald’s omega (ω) reliabilities for the aggregated data were 0.866 for team leadership, 0.792 for perceived support, 0.748 for psychological safety, and 0.830 for learning behavior, well above the frequently cited cutoff of 0.70 [38].

**Table 1:** Overview of Variables and Measures

Model Construct	Variable	Measure	Measure Type	Data Source
Control Variables	Staff-to-bed ratio	Total number of hospital staff divided by total number of hospital beds	Continuous	Administrative Dataset
	Rural	Indicator of whether a hospital is rural or other (e.g., urban or peri-urban)	Binary: 0 = N; 1 = Y	Administrative Dataset
	QI team size	Average response to “Estimate the number of people who are on your QI team”	Continuous	QI Team Questionnaire
	Average age	Average response to “Age (years)”	Continuous	QI Team Questionnaire
	Hospital staff size	Total number of staff that work at the hospital	Continuous	Administrative Dataset
Predictors				
Latent Variable: Perceived Support	Support-1	It is easy for my team to obtain expert assistance when something comes up that we don’t know how to handle.	Aggregate (7-pt. Likert)	QI Team Questionnaire
	Support-2	My team has the financial resources it needs carry out QI activities.	Aggregate (7-pt. Likert)	QI Team Questionnaire
	Support-3	Leaders at my facility strongly support the work of my team.	Aggregate (7-pt. Likert)	QI Team Questionnaire
	Support-4	Leaders at my facility have made QI a high priority.	Aggregate (7-pt. Likert)	QI Team Questionnaire
Latent Variable: Team Leadership	Lead-1	There is a person on my team who initiates meetings to discuss the team’s progress.	Aggregate (7-pt. Likert)	QI Team Questionnaire
	Lead-2	There is a person on my team who is available for consultation on problems.	Aggregate (7-pt. Likert)	QI Team Questionnaire
	Lead-3	There is a person on my team who provides feedback on team member performance, identifying strengths and weaknesses.	Aggregate (7-pt. Likert)	QI Team Questionnaire
Latent Variable: Psychological Safety	Safety-1	All members of the team are encouraged to speak up and ask questions, regardless of their position in the organization.	Aggregate (7-pt. Likert)	QI Team Questionnaire
	Safety-2	We appreciate and build upon our individual differences.	Aggregate (7-pt. Likert)	QI Team Questionnaire
	Safety-3	It is DIFFICULT to ask other members of my team for help.	Aggregate (7-pt. Likert)	QI Team Questionnaire
	Safety-4	People on this team sometimes REJECT OTHERS for being different.	Aggregate (7-pt. Likert)	QI Team Questionnaire
	Safety-5	If you make a mistake on my team, it is often HELD AGAINST YOU.	Aggregate (7-pt. Likert)	QI Team Questionnaire
Latent Variable: Learning Behavior	Learn-1	My team openly discusses mistakes so that we can learn from them.	Aggregate (7-pt. Likert)	QI Team Questionnaire
	Learn-2	We regularly take time to learn ways to improve how we do our work.	Aggregate (7-pt. Likert)	QI Team Questionnaire
	Learn-3	My team always takes time to stop and reflect on our work.	Aggregate (7-pt. Likert)	QI Team Questionnaire
Team Effectiveness				
Latent Variable: QI Implementation	Perform-1	This team meets or exceeds the expectations of Project Fives Alive.	7-pt. Likert	Project Officer Survey
	Perform-2	This team does superb work.	7-pt. Likert	Project Officer Survey
	Perform-3	This team keeps getting better and better.	7-pt. Likert	Project Officer Survey
	QI Practice-1	The team evaluates reasons for variation in how work is carried out.	7-pt. Likert	Project Officer Survey
	QI Practice-2	The team has made an actual change in the way some aspect of work gets done.	7-pt. Likert	Project Officer Survey
	QI Practice-3	The team meets frequently to work on quality improvement.	7-pt. Likert	Project Officer Survey

We conducted confirmatory factor analyses (CFAs) to evaluate the proposed measurement model. The CFAs revealed good or acceptable model fit (Table 2). We calculated measures of interrater reliability (R_wg(j)_) and intraclass correlation (ICC_1_) to justify our decision to aggregate data to the group level (Table 3). The lowest R_wg(j)_ value was 0.745 for team leadership and the lowest average ICC_1_ was for 0.149 for learning behavior. Collectively, the R_wg(j)_ and ICC_1_ values indicate moderate to strong agreement at the group level [39–41].

**Table 2:** Fit Statistics for Confirmatory Factor Analyses and Structural Models

Model Description	Number of:				DF	χ^2^ *(p-value)* ^a^	RMSEA *(Pr ≤ 0.05)*	CFI	BIC_s_^b^
	Observations (Teams)	Latent Variables	Observed Variables	Free Parameters					
CFA: LEAD with SUPPORT	127	2	7	23	12	17.0 *(0.149)*	0.057 *(0.376)*	0.983	−41.1
CFA: SAFETY with LEARN	127	2	8	26	18	42.8 *(0.001)*	0.104 *(0.016)*	0.919	−44.4
CFA: QI Implementation	122	1	6	19	8	5.7 *(0.677)*	0.000 *(0.828)*	1.000	−32.7
Structural Model: Complete Mediation^c^	122	5	26	113	264	403.7 *(< 0.001)*	0.066 *(0.025)*	0.920	− 864.6
Structural Model: Complete Mediation^d^	122	5	26	116	261	371.7 *(< 0.001)*	0.059 *(0.142)*	0.936	−882.1
Structural Model: Partial Mediation^d^	122	5	26	121	256	360.8 *(< 0.001)*	0.058 *(0.175)*	0.940	−869.0

Notes: χ^2^ is more likely to reject fit with larger N and more variables

RMSEA is more likely to reject fit with smaller N and lower DF

BIC_s_ < 0 indicates good model fit. When comparing similar models, the more negative BIC indicates better fit

^a^ Scaled χ^2^ [56]

^b^ Schwarz BIC [47]

^c^ Model includes no correlated errors

^d^ Model includes three correlated errors based on a priori expectations

**Table 3:** Team-Level Descriptive Statistics

Model Construct	Variable	Mean *(SD)*	Min	Max	Obs	R_wg(J)_ *(distribution)*	ICC_1_	ω reliability
Control Variables	Hospital staff size	252.3 *(177.2)*	43	1100	113	n/a	n/a	n/a
	Average age	34.13 *(4.86)*	26.75	48.33	121			
	QI team size^a^	9.10 *(3.47)*	4.00	24.40	121			
	Rural hospital	23.7%	*n/a*	*n/a*	122			
	Staff-to-bed ratio	3.16 *(2.69)*	0.54	21.95	110			
Perceived Support	Support-1	5.29 *(1.01)*	2.0	7.0	122	0.605 *(slightly skewed)*	0.354	0.792
	Support-2	3.50 *(1.40)*	1.0	6.3	122			
	Support-3	5.01 *(1.29)*	1.3	7.0	122			
	Support-4	4.83 *(1.29)*	1.7	7.0	122			
Team Leadership	Lead-1	5.83 *(0.78)*	3.5	7.0	122	0.587 *(moderately skewed)*	0.213	0.866
	Lead-2	5.66 *(0.92)*	1.7	7.0	122			
	Lead-3	5.10 *(1.05)*	1.7	7.0	122			
Psychological Safety	Safety-1	6.26 *(0.53)*	4.3	7.0	122	0.745 *(heavily skewed)*	0.153	0.748
	Safety-2	6.10 *(0.46)*	4.3	7.0	122			
	Safety-3^b^	6.25 *(0.63)*	3.7	7.0	122			
	Safety-4^b^	6.35 *(0.57)*	4.0	7.0	122			
	Safety-5^b^	6.44 *(0.51)*	4.0	7.0	122			
Learning Behavior	Learn-1	5.96 *(0.65)*	3.5	7.0	122	0.718 *(moderately skewed)*	0.149	0.830
	Learn-2	5.54 *(0.73)*	3.0	7.0	122			
	Learn-3	5.37 *(0.77)*	2.8	7.0	122			
QI Implementation	Perform-1	5.19 *(1.03)*	2.0	7.0	122	n/a	n/a	0.912
	Perform-2	5.54 *(1.19)*	1.0	7.0	122			
	Perform-3	4.33 *(1.42)*	1.0	7.0	122			
	QI Practice-1	4.48 *(1.61)*	1.0	7.0	122			
	QI Practice-2	4.54 *(1.46)*	1.0	7.0	122			
	QI Practice-3	4.63 *(1.59)*	1.0	7.0	122			

*N* = 122 teams

^a^QI team size refers to the average of the reported number of people on each team

^b^Scores for negatively-worded items were reverse coded (e.g., 1 = 7

#### QI implementation

QI implementation, the key outcome measure, refers to the intensity and fidelity with which teams implement QI methods. Ratings of QI implementation came from project officer surveys, which included 13 questions on the performance of QI teams. Eight project officers completed surveys evaluating 122 teams. Project officers were instructed to complete the surveys during site visits with each team prior to the fourth round of learning sessions. As outsiders who each worked closely with a subset of the QI teams, project officers were well positioned to evaluate team performance.

Because there is no well-established instrument for evaluating the QI implementation, we combined a three-item team performance scale with three questions about a team’s QI activity (Table 1) [13, 42, 43]. All six items used a 7-point Likert scale from “completely disagree” to “completely agree.” For the performance items, project officers were instructed to “think about… how well team members work together and how effectively the team implements QI methods.” Because the performance and QI activity items all referenced QI and used the same response scale, we combined them into a single latent variable. A CFA revealed excellent model fit (Table 2) and a high ω reliability of 0.912.

#### Control variables

We controlled for the average age of respondents, the average reported QI team size, rural location, staff size, and staff-to-bed ratio. Control variables came from two sources (Table 1). First, the QI team questionnaire included questions about demographics and team composition. Second, the hospital administrative dataset provided information on the type, location, size, and staffing of each hospital. In most cases, the project officers contacted hospital administrators by phone to collect this information.

### Statistical analyses

We tested our hypotheses using structural equation modeling (SEM). SEM consists of simultaneous multivariate regressions and allows for the estimation of unobserved, latent variables using shared variance from observed variables. This eliminates bias from measurement error [35]. SEM also allows researchers to simultaneously estimate multiple paths and test of direct, indirect, and total effects [35].

We conducted the analysis at the team level. A multilevel model was also developed but demonstrated issues with convergence, likely due to an average team size under 10, unequal ICC_1_ values, or ICC_1_ values under 0.25 [44]. Due to issues like these, aggregation remains the typical approach when dealing with group-level data collected across individuals. Although the R_wg(j)_ and ICC_1_ values in Table 3 provide strong evidence in favor of aggregation, this can still produce biased standard errors. However, the bias for our standard error parameters is likely low because all survey questions referenced the group and because the factors should operate similarly at both the individual and team level of analysis [44, 45]. We conducted the analysis in Mplus (v 7.4) using a maximum likelihood estimator with robust standard errors clustered by region. Although maximum likelihood estimation works best with continuous data, Likert-type questions approximate continuous data when they have a response scale with six or more questions and the distributions are not highly skewed [46].

The first step in analyzing structural equation models is evaluating model fit. Model fit provides an indication of how closely observed data match expected data given a specified model. Poorly fitting models may provide biased results. We evaluated fit using the scaled chi-squared (χ^2^) value, comparative fit index (CFI), and root mean squared error of approximation (RMSEA). Good fit is indicated by an insignificant χ^2^ value, CFI greater than 0.95, and RMSEA less than 0.05 [35, 47, 48]. We initially tested a model of complete mediation. We tested improvements in model fit by adding three correlated errors based on a priori expectations about relationships between items. We also compared the results and fit of the model of complete mediation to a model of partial mediation.

After we achieved a well-fitting model, we tested the significance of all standardized estimates. We also estimated and tested standardized indirect and total effects. Standardized parameters are transformations of unstandardized regression coefficients that remove scaling and better allow for comparison of effects across parameters (β_stdyx_ = β*σ_x_/σ_y_) [49]. Indirect effects are the products of regression coefficients along specified indirect paths [49]. Main results are also described using the original scale to indicate the meaning of the effect size.

## Results

### Study population and descriptive statistics

A total of 141 hospital-based QI teams participated in PFA. Of these teams, four teams did not attend the fourth round of learning sessions and did not complete the QI team questionnaire. Questionnaires were completed by 602 individuals from the remaining 137 teams. We excluded: teams that were given outdated survey forms; teams that were missing project officer surveys; individuals who exhibited a strong tendency towards response sets by consistently answering positively- and negatively-worded questions similarly; and individual responses that were missing entirely for a factor in this study. The final analytical sample included 490 individuals from 122 QI teams for an average of 4.02 respondents per team. Fourteen additional teams were missing data on one or more variables from the hospital administrative dataset. However, SEM handles missing data well; we chose to include these teams in the analysis since they were only missing data for control variables. Because we aggregated data to the team level, none of the teams were missing data for items from the QI team questionnaires. Table 3 provides descriptive statistics for the 122 teams included in the analysis.

### SEM model fit

We first tested a model of complete mediation. The fit indices for this model indicated “acceptable” model fit; CFI was 0.920 and RMSEA was 0.066 with a significant *p*-value (Table 2). We relaxed the model by allowing correlated errors for the following three pairs of items (Table 1):

- Support-3 and Support-4 because the items had highly similar meaning and wording;
- Safety-1 and Safety-2 because these two items were positively-worded, whereas the remaining three items for psychological safety were negatively-worded;
- QI Practice-1 and QI Practice-2 because these questions both came from Lemieux-Charles et al. [39] and were the most technical of all the QI implementation questions.

Adding these correlated errors produced a CFI of 0.936 and RMSEA of 0.059 with an insignificant *p*-value, indicating improvement over the basic model. The Schwarz Bayesian information criteria (BIC_s_) can be used to further compare the fit of two similar models; the more negative the BIC_s_, the better the model fit [50]. The difference between the base model and the model with three correlated errors was − 15, providing “very strong” evidence in favor of the model with the correlated errors [50].

Because complete mediation is often an unrealistic expectation, we also evaluated the fit of a model of partial mediation with the same correlated errors (Table 2). The model of partial mediation allows all latent variables to act on subsequent latent variables, freeing up paths otherwise restricted to zero. The model of partial mediation produced a CFI of 0.940 and RMSEA of 0.058 with an insignificant p-value. Although the model of partial mediation appears to have slightly improved fit, the BIC_s_ for the model of partial mediation was − 869.0 compared to − 882.1 for the model of complete mediation, providing “very strong” evidence in favor of complete mediation [50].

**Fig. 1:**
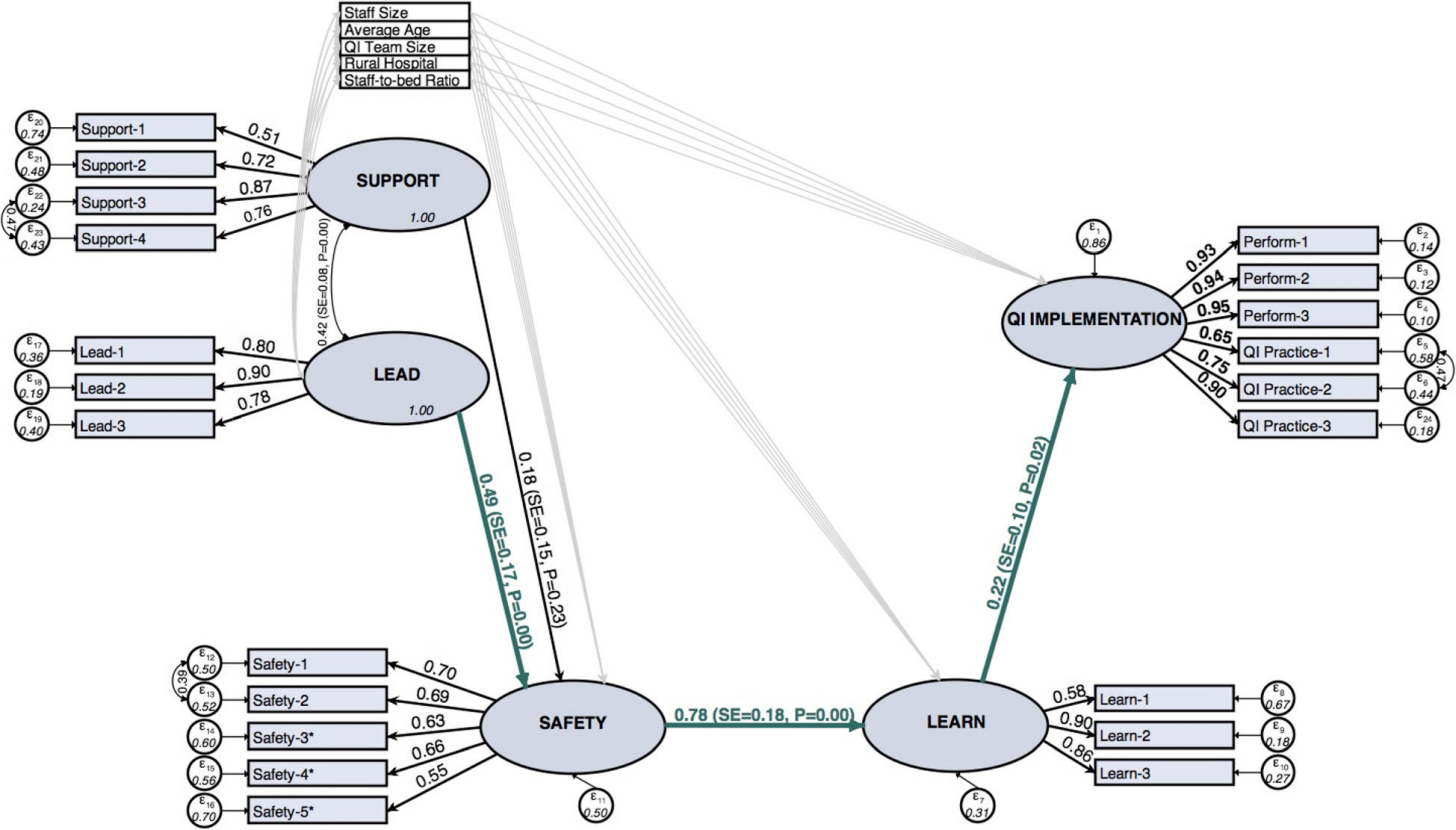
Standardized SEM results for the model of complete mediation. Note: Paths and Correlations for control variables were included in the model, but the estimates are not shown here (see Table 4). * Scores for negatively-worded items were reverse coded

We present results from the model of complete mediation (Fig. 1). Additional improvements in fit may be possible, but they would not be based on theory. Additionally, the fit indices for the model of complete mediation indicate “acceptable” fit and do not suggest any major misspecifications.

### SEM results

Because the Likert-type survey items have difference means and variances, we present the correlation between variables (standardized estimates) in Fig. 1 and Table 4. Learning behavior had a significant positive effect on QI implementation (β = 0.22, *p* = 0.02) (Table 4), psychological safety had a significant direct effect on learning behavior (β = 0.78, *p* < 0.001), and psychological safety had a significant indirect effect on QI implementation through learning behavior (β = 0.171, *p* = 0.001). This was also the dominant pathway in the model of partial mediation. Although psychological safety had a strong positive effect on learning behavior and team leadership had a strong positive effect on psychological safety (β = 0.49, *p* < 0.001), the indirect effect of team leadership on psychological safety was only significant at α = 0.10 (β = 0.38, *p* = 0.068). Finally, perceived support for teamwork was not significantly associated with any factor except team leadership. After converting the results back to the original 7-point Likert scale, a 1-point change in the average team rating of team leadership was associated with a 0.492-point (*p* = 0.003) increase in the response to the average team rating of psychological safety. A 1-point change in the average team rating of psychological safety was associated with a 0.779-point (*p* < 0.001) change in the average team rating of learning behavior. Finally, a 1-point change in the average team rating of learning behavior was associated with a 0.876-point (*p* = 0.012) change in the project officer rating of QI implementation.

**Table 4:** Standardized SEM Results

Factor	Partial Mediation		Complete Mediation	
	Standardized Estimates	Standard Error	Standardized Estimates	Standard Error
QI Implementation				
on Learning Behavior	0.284*	0.147	0.220**	0.097
on Psychological Safety	−0.113	0.138		
on Team Leadership	−0.064	0.073		
on Perceived Support	−0.156	0.149		
*on Hospital staff size*	*0.016*	*0.183*	*0.003*	*0.069*
*on Average age*	−0.150*	*0.079*	−0.144**	*0.070*
*on QI team size*	*0.134*	*0.109*	*0.111*	*0.108*
*on Rural hospital^*	*−0.029*	*0.183*	*−0.039*	*0.181*
*on Staff-to-bed ratio*	0.163*	*0.083*	0.174***	*0.065*
Learning Behavior				
on Psychological Safety	0.530**	0.233	0.779***	0.184
on Team Leadership	0.313*	0.170		
on Perceived Support	0.000	0.070		
*on Hospital staff size*	*−0.115*	*0.118*	*−0.035*	*0.111*
*on Average age*	*−0.013*	*0.066*	*−0.018*	*0.065*
*on QI team size*	*0.040*	*0.131*	*0.040*	*0.148*
*on Rural hospital^*	*0.010*	*0.074*	*0.070*	*0.109*
*on Staff-to-bed ratio*	*0.083*	*0.063*	*0.092*	*0.126*
Psychological Safety				
on Team Leadership	0.386	0.175	0.492***	0.167
on Perceived Support	0.201	0.142	0.184	0.152
*on Hospital staff size*	*−0.198*	*0.176*	*−0.227*	*0.142*
*on Average age*	*0.046*	*0.059*	*0.044*	*0.075*
*on QI team size*	0.201***	*0.038*	0.187***	*0.045*
*on Rural hospital^*	*0.003*	*0.093*	*−0.018*	*0.087*
*on Staff-to-bed ratio*	*−0.002*	*0.143*	*0.013*	*0.152*
Team Leadership				
with Perceived Support	0.414***	0.089	0.415***	0.081
*with Hospital staff size*	*−0.020*	*0.083*	*−0.018*	*0.083*
*with Average age*	*−0.005*	*0.077*	*−0.007*	*0.077*
*with QI team size*	0.301***	*0.086*	0.298***	*0.086*
*with Rural hospital^*	0.311***	*0.035*	0.309***	*0.036*
*with Staff-to-bed ratio*	0.186*	*0.109*	0.185*	*0.108*
Perceived Support				
*with Hospital staff size*	−0.141**	*0.060*	−0.137**	*0.059*
*with Average age*	*0.072*	*0.118*	*0.072*	*0.118*
*with QI team size*	*0.086*	*0.148*	*0.088*	*0.147*
*with Rural hospital^*	0.125***	*0.028*	0.127***	*0.031*
*with Staff-to-bed ratio*	*0.087*	*0.058*	*0.091*	*0.058*

*N* = 122; fit statistics shown in Table 2

^ Compared to urban hospitals

*Statistically significant at *p* < 0.10

**Statistically significant at *p* < 0.05

***Statistically significant at *p* < 0.01

Standardized estimates indicate the change in y associated with a one standard deviation change in x

We controlled for hospital staff size, average respondent age, QI team size, rural location, and staff-to-bed ratio. As expected, these contextual factors had stronger effects on antecedent conditions than factors from the other domains. However, average respondent age and staff-to-bed ratio both had significant effects on QI implementation. Average respondent age was negatively associated with QI implementation (β = − 0.144, *p* = 0.040) and staff-to-bed ratio had a strong positive association with QI implementation (β = 0.174, *p* = 0.008). Although the majority of the effect was direct, staff-to-bed ratio also had a small, but significant, positive indirect effect on QI implementation. Overall, the model of complete mediation explained 14.1% of the variance in QI implementation.

## Discusssion

This study analyzed factors associated with the implementation of QI methods by hospital-based teams working to reduce under-5 mortality in Ghana. The results provide strong support for hypothesis-1, showing that learning behavior mediates a positive relationship between psychological safety and QI implementation in newly developed QI teams working in low-income countries. We only find moderate support for hypothesis-2; psychological safety also appears to mediate the effect of team leadership on learning behavior, however, team leadership may also have a direct effect on learning behavior or other indirect effects not explained in our model. The results do not support hypothesis-3. Perceived support had neither a significant direct or indirect effect on psychological safety or learning behavior. However, perceived support may influence learning behavior through other factors not included in this analysis, like team efficacy [13].

These findings suggest potential leverage points that could be targeted when teams exhibit low QI implementation effectiveness. In particular, the team development process should emphasize team leadership. In addition to training teams on QI methods, leaders should be trained in concepts like psychological safety, conflict management, and motivation. This may be even more important when implementing team-based activities like QI in new settings with significant cultural differences. Furthermore, because team members are typically acutely aware of leader behavior, the influence of leaders may be unintentional [51]. Leaders should consider how all of their actions affect the team.

Some QI teams may not benefit from interventions to improve psychological safety. For example, some teams may exhibit high implementation effectiveness, but fail to produce clinical improvements. These teams may experience barriers beyond the control of the team. They might produce useful information, but facility managers could discourage change or otherwise impede progress. On the other hand, other factors, such as limited time, resources, or training, could lead to poor implementation of QI methods. In these cases, implementation effectiveness may remain low even if psychological safety is high. Periodic evaluations could help identify issues and reveal which teams would benefit most from an intervention.

Although the roles of psychological safety and learning behavior have been explored in healthcare, this is the first study that we know of to use SEM for the analysis [29, 32]. In addition to controlling for measurement error, SEM allowed us to estimate the magnitude and significance of indirect effects and to test overall model fit. The more parsimonious model of complete mediation provided acceptable model fit and was favored over the model of partial mediation, indicating that the observed data reasonably matched the hypothesized structural relationships. Furthermore, the model of partial mediation only explained a slightly larger percentage of the variance of QI implementation. Collectively, this provides strong support for the model of work-team learning in a way not yet shown empirically.

This is also the first quantitative study to explore team-level predictors of QI implementation in lower-income countries. Additionally, whereas Tucker et al. linked team learning to perceived implementation success, a measure of the degree to which changes were perceived as improving care, we used measures of actual QI implementation [29]. This is a key distinction as implementation effectiveness is a key determinant of innovation effectiveness [15]. Mixed results from evaluations of large-scale QI interventions may be explained by differences in the implementation [4, 52]. Understanding how well teams implement QI could help facilitators, coaches, and others find effective ways to support local QI teams.

This study has implications for future research. We found evidence that it may take time for team members to develop shared appraisals of the team and that psychological safety may arise as an emergent team state [18, 53]. A CFA of psychological safety from a questionnaire distributed 2–4 months after teams were developed (compared to 16–18 months as described in this study) revealed poor model fit. This suggests team members may have a poor understanding of team psychological safety early after team development. Future work is needed to evaluate how and when members develop a shared understanding of psychological safety and how psychological safety changes over time. If perceptions of psychological safety are resistant to change, the period immediately after team formation may be a critical time to established a psychologically safe environment.

This study builds on existing literature and suggests that the model of work team learning extends beyond high income countries and applies to lower-income countries as well. However, these results are based on teams with a moderate average age (34.1 among survey respondents), an average QI team size of 9.1 members, a staff-to-bed ratio of 3.16, and an average hospital staff size of 252 (range = 43 to 1100). Additional work is also needed to evaluate the impact of contextual factors. For example, there may be a threshold in the minimum team size at which team psychological safety and team learning behavior become critically important for team effectiveness. The role of these factors also likely varies to some degree between temporary and permanent teams.

Psychological safety may influence sustainability. Researchers now recognize a cyclical causal feedback loop where past performance influences future performance [54, 55]. As a result, low early psychological safety may limit future implementation effectiveness and overall sustainability of efforts. Indeed, PFA project officers have suggested that some QI teams are held back because they have never experienced success.

### Limitations

Although this study makes several important contributions, the study also has several limitations. First, relationships between team-level factors derived from the QI team questionnaire are subject to common method bias. Likewise, social desirability of resp. may influence responses. Although the effect of social desirability and common-method bias may not be null, QI implementation was rated by external observers which helps address these concerns. Second, we are unable to claim causation from an observational study using survey data. Additionally, the results are only valid to the degree that the latent factors actually capture the concepts of interest. Third, aggregating the data to the team level results in a less than ideal sample size for SEM, reducing our power and ability to detect model misspecifications. However, our model was locally and globally identified and our final sample exceeded the number of free parameters. A multilevel model would have been ideal, but we experienced issues with model convergence. Estimating the model at the group level helped resolve convergence issues. However, we were forced reduce the overall number of free parameters by removing items from latent factors and dropping potential control variables. Finally, the results may only be generalizable to situations involving the development of QI teams in settings like Ghana.

## Conclusion

We find convincing evidence that psychological safety and learning behavior are key determinants of QI implementation in lower-income countries, a non-traditional setting for organizational research. Consistent with other research, this study also demonstrates that leaders play a critical role in establishing a climate of psychologically safety that supports effective teamwork and learning behavior. This may be especially important as lower-income countries work to develop a mature and effective healthcare workforce.

## Data Availability

The datasets analyzed in the current study are available from the corresponding author upon reasonable request after approval from program leadership/oversight.

## Abbreviations

BIC_s_: `Schwarz Bayesian information criteria
CFA: Confirmatory factor analysis
CFI: Comparative fit index
ICC_1_: Intraclass correlation
PFA: Project Fives Alive
QI: Quality improvement
RMSEA: Root mean squared error of approximation
RWG_(J)_: Interrater reliability for a scale with j items
SEM: Structural equation modeling
